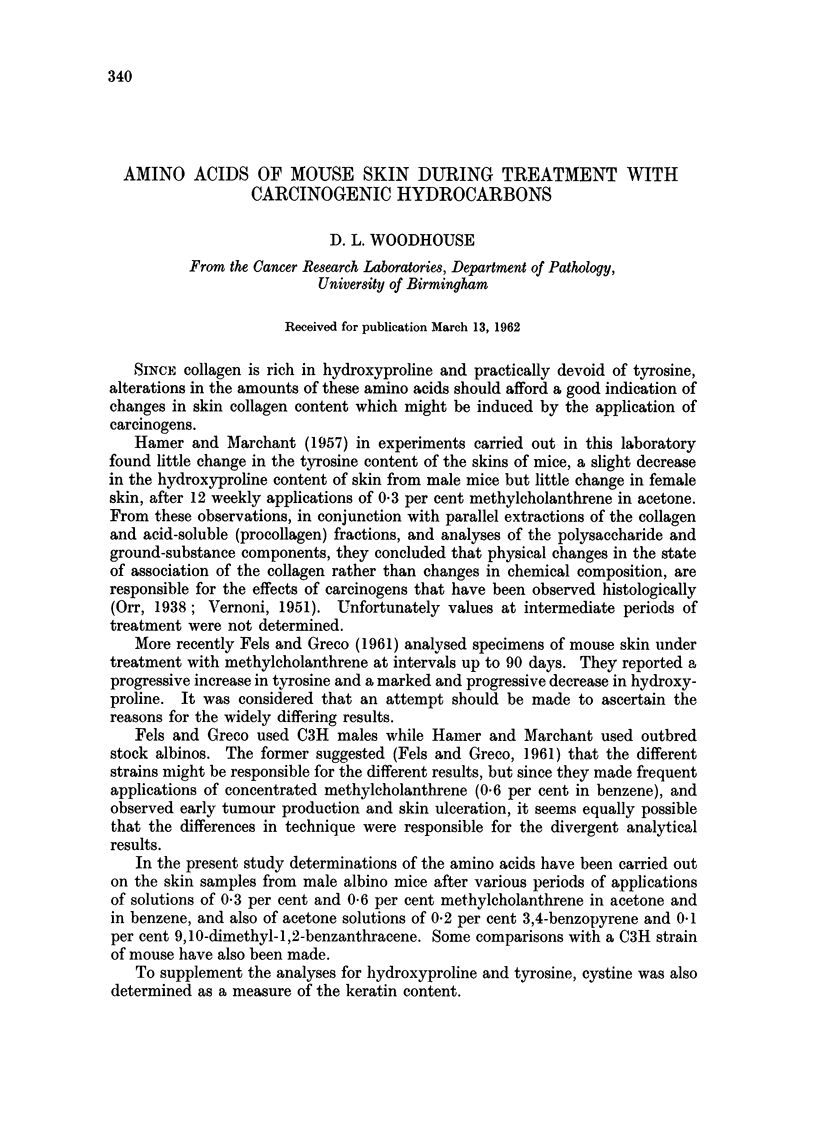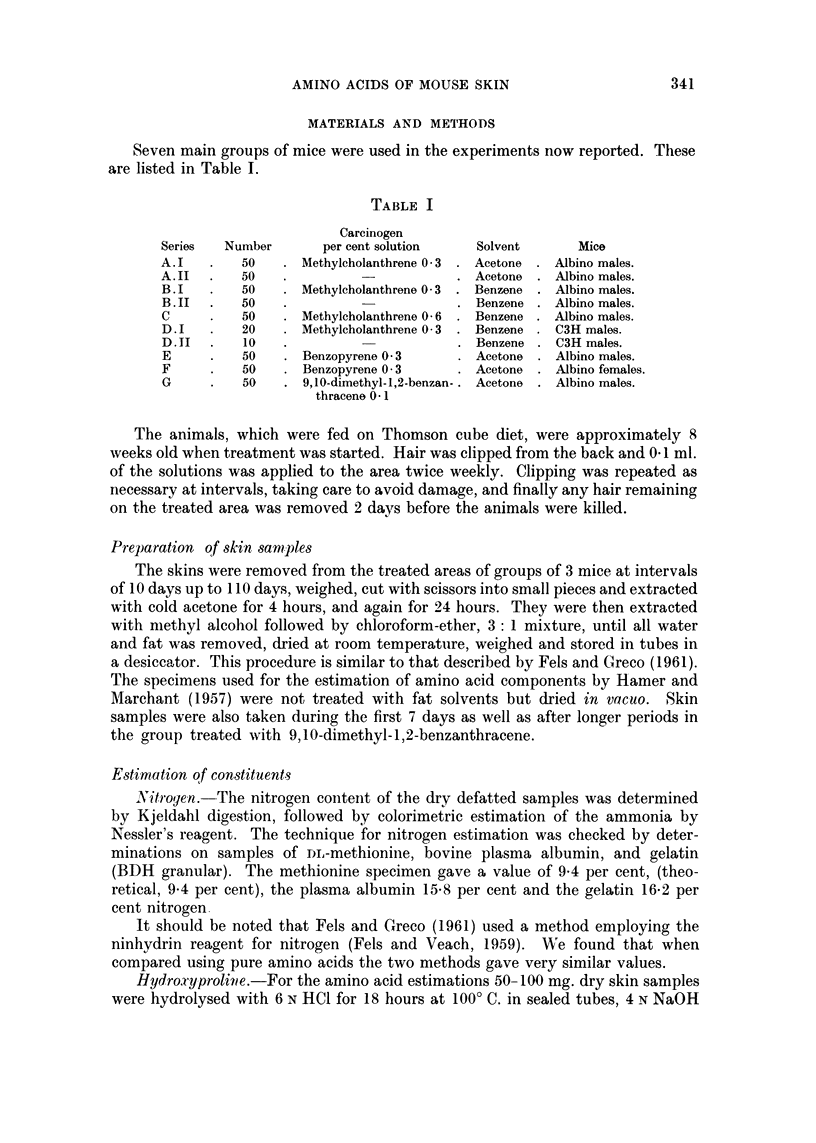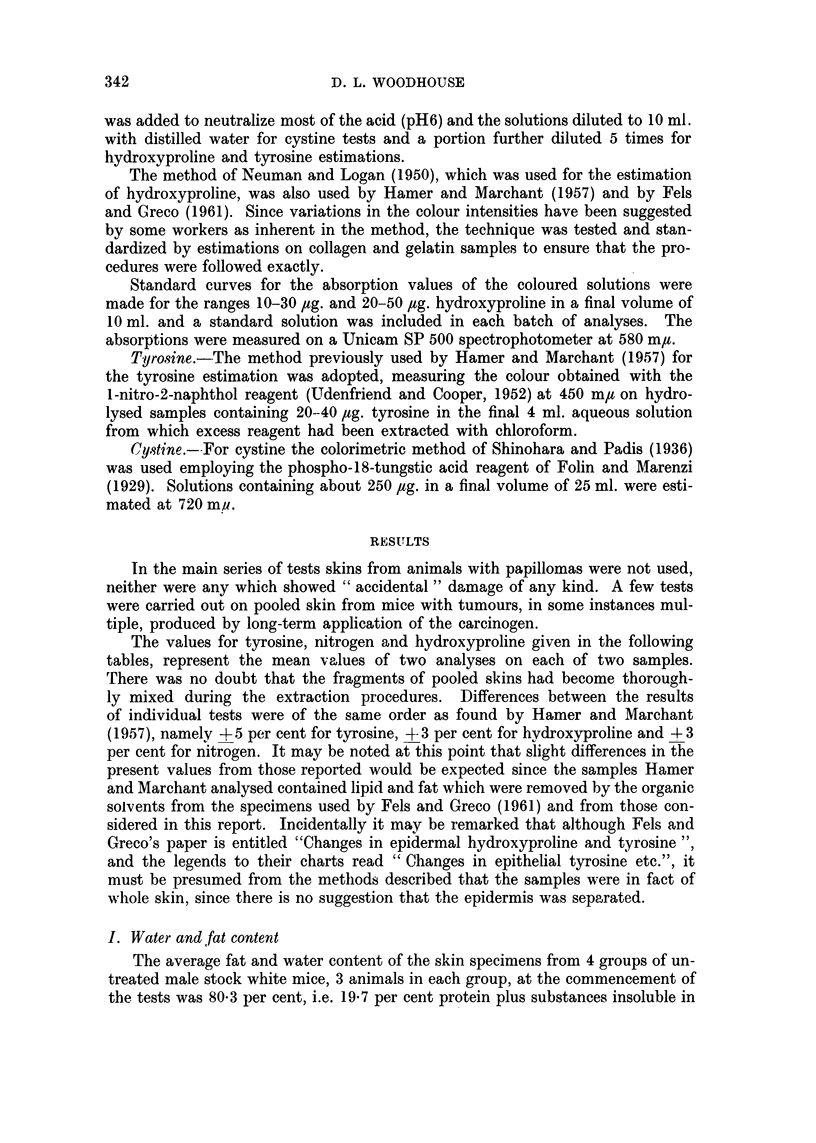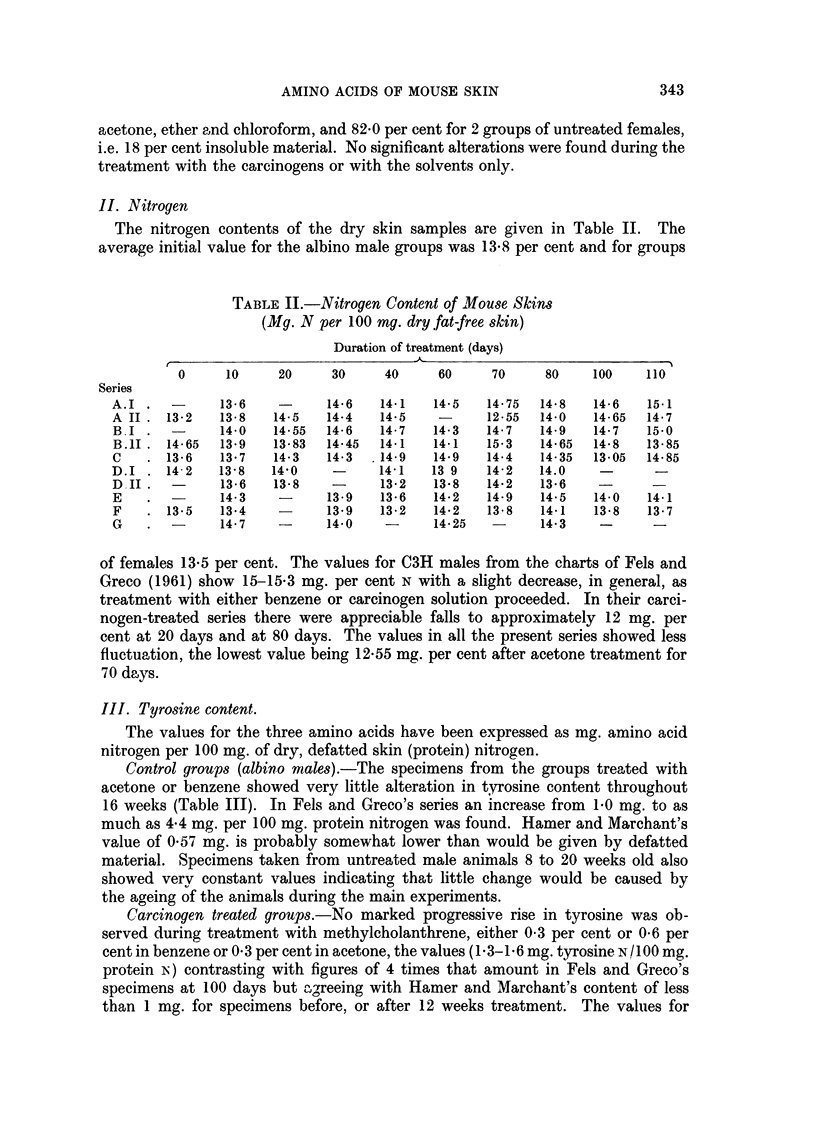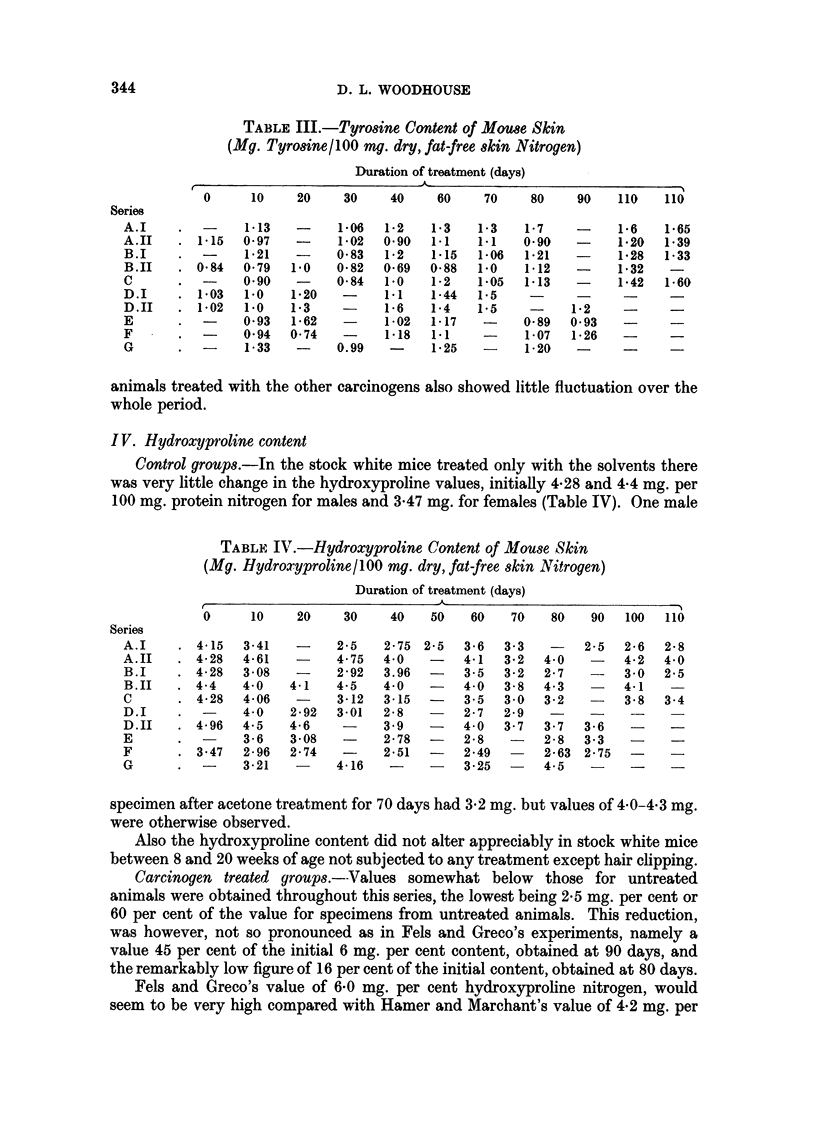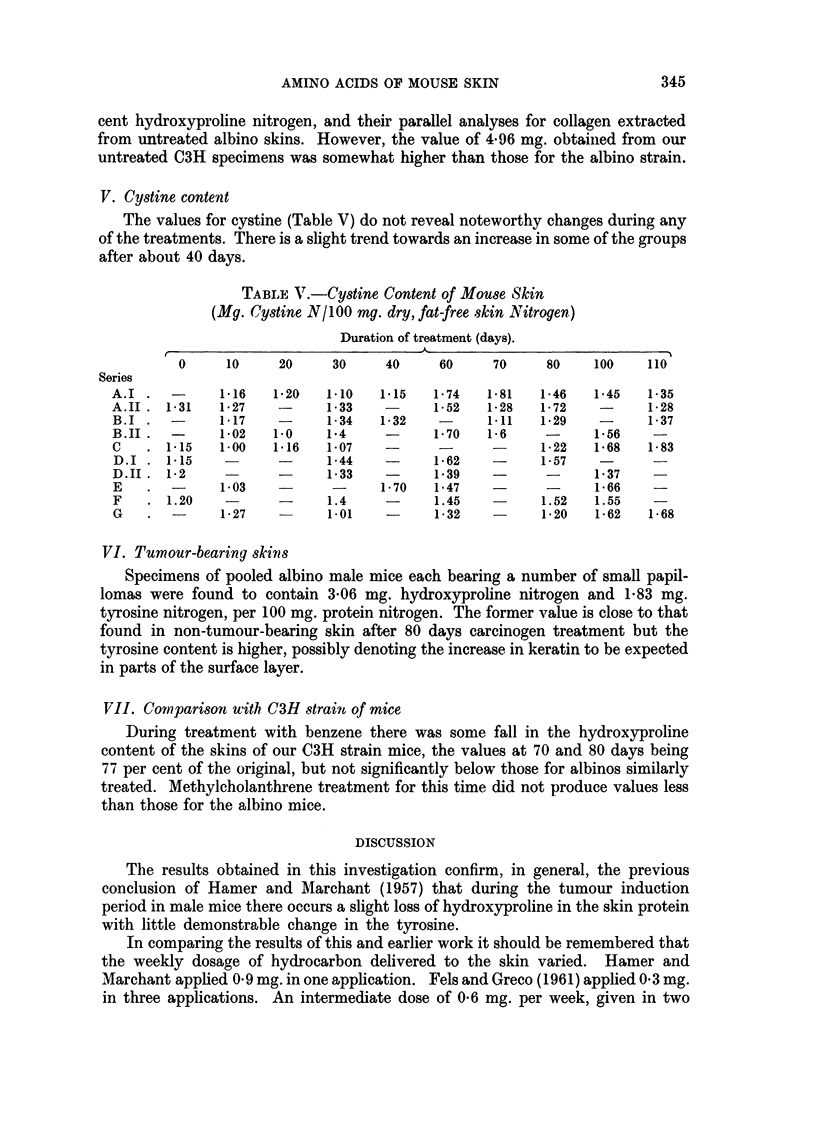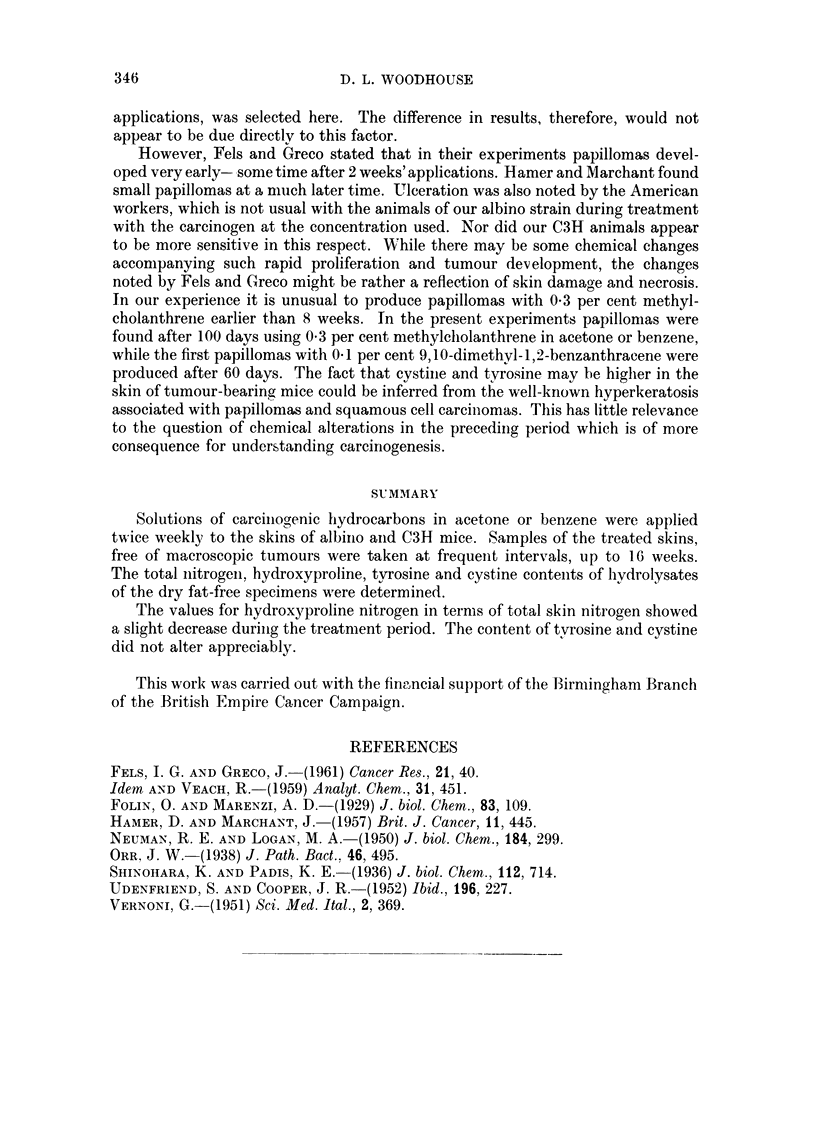# Amino Acids of Mouse Skin During Treatment with Carcinogenic Hydrocarbons

**DOI:** 10.1038/bjc.1962.39

**Published:** 1962-06

**Authors:** D. L. Woodhouse


### AMINO ACIDS OF MOUSE SKIN DURING TREATMENT WITH CARCINOGENIC HYDROCARBONS

D. L. WOODHOUSE

*From the Cancer Research Laboratories, Department of Pathology, University of Birmingham*

Received for publication March 13, 1962

SINCE collagen is rich in hydroxyproline and practically devoid of tyrosine, alterations in the amounts of these amino acids should afford a good indication of changes in skin collagen content which might be induced by the application of carcinogens.

Hamer and Marchant (1957) in experiments carried out in this laboratory found little change in the tyrosine content of the skins of mice, a slight decrease in the hydroxyproline content of skin from male mice but little change in female skin, after 12 weekly applications of 0·3 per cent methylcholanthrene in acetone. From these observations, in conjunction with parallel extractions of the collagen and acid-soluble (procollagen) fractions, and analyses of the polysaccharide and ground-substance components, they concluded that physical changes in the state of association of the collagen rather than changes in chemical composition, are responsible for the effects of carcinogens that have been observed histologically (Orr, 1938; Vernoni, 1951). Unfortunately values at intermediate periods of treatment were not determined.

More recently Fels and Greco (1961) analysed specimens of mouse skin under treatment with methylcholanthrene at intervals up to 90 days. They reported a progressive increase in tyrosine and a marked and progressive decrease in hydroxyproline. It was considered that an attempt should be made to ascertain the reasons for the widely differing results.

Fels and Greco used C3H males while Hamer and Marchant used outbred stock albinos. The former suggested (Fels and Greco, 1961) that the different strains might be responsible for the different results, but since they made frequent applications of concentrated methylcholanthrene (0·6 per cent in benzene), and observed early tumour production and skin ulceration, it seems equally possible that the differences in technique were responsible for the divergent analytical results.

In the present study determinations of the amino acids have been carried out on the skin samples from male albino mice after various periods of applications of solutions of 0·3 per cent and 0·6 per cent methylcholanthrene in acetone and in benzene, and also of acetone solutions of 0·2 per cent 3,4-benzopyrene and 0·1 per cent 9,10-dimethyl-1,2-benzanthracene. Some comparisons with a C3H strain of mouse have also been made.

To supplement the analyses for hydroxyproline and tyrosine, cystine was also determined as a measure of the keratin content.

### MATERIALS AND METHODS

Seven main groups of mice were used in the experiments now reported. These are listed in Table I.

TABLE I

| Series | Number | Carcinogen per cent solution | Solvent | Mice |
|---|---|---|---|---|
| A.I | 50 | Methylcholanthrene 0·3 | Acetone | Albino males. |
| A.II | 50 | — | Acetone | Albino males. |
| B.I | 50 | Methylcholanthrene 0·3 | Benzene | Albino males. |
| B.II | 50 | — | Benzene | Albino males. |
| C | 50 | Methylcholanthrene 0·6 | Benzene | Albino males. |
| D.I | 20 | Methylcholanthrene 0·3 | Benzene | C3H males. |
| D.II | 10 | — | Benzene | C3H males. |
| E | 50 | Benzopyrene 0·3 | Acetone | Albino males. |
| F | 50 | Benzopyrene 0·3 | Acetone | Albino females. |
| G | 50 | 9,10-dimethyl-1,2-benzanthracene 0·1 | Acetone | Albino males. |

The animals, which were fed on Thomson cube diet, were approximately 8 weeks old when treatment was started. Hair was clipped from the back and 0·1 ml. of the solutions was applied to the area twice weekly. Clipping was repeated as necessary at intervals, taking care to avoid damage, and finally any hair remaining on the treated area was removed 2 days before the animals were killed.

### *Preparation of skin samples*

The skins were removed from the treated areas of groups of 3 mice at intervals of 10 days up to 110 days, weighed, cut with scissors into small pieces and extracted with cold acetone for 4 hours, and again for 24 hours. They were then extracted with methyl alcohol followed by chloroform-ether, 3 : 1 mixture, until all water and fat was removed, dried at room temperature, weighed and stored in tubes in a desiccator. This procedure is similar to that described by Fels and Greco (1961). The specimens used for the estimation of amino acid components by Hamer and Marchant (1957) were not treated with fat solvents but dried *in vacuo*. Skin samples were also taken during the first 7 days as well as after longer periods in the group treated with 9,10-dimethyl-1,2-benzanthracene.

### *Estimation of constituents*

*Nitrogen.*—The nitrogen content of the dry defatted samples was determined by Kjeldahl digestion, followed by colorimetric estimation of the ammonia by Nessler's reagent. The technique for nitrogen estimation was checked by determinations on samples of DL-methionine, bovine plasma albumin, and gelatin (BDH granular). The methionine specimen gave a value of 9·4 per cent, (theoretical, 9·4 per cent), the plasma albumin 15·8 per cent and the gelatin 16·2 per cent nitrogen.

It should be noted that Fels and Greco (1961) used a method employing the ninhydrin reagent for nitrogen (Fels and Veach, 1959). We found that when compared using pure amino acids the two methods gave very similar values.

*Hydroxyproline.*—For the amino acid estimations 50–100 mg. dry skin samples were hydrolysed with 6 N HCl for 18 hours at 100° C. in sealed tubes, 4 N NaOH

was added to neutralize most of the acid (pH6) and the solutions diluted to 10 ml. with distilled water for cystine tests and a portion further diluted 5 times for hydroxyproline and tyrosine estimations.

The method of Neuman and Logan (1950), which was used for the estimation of hydroxyproline, was also used by Hamer and Marchant (1957) and by Fels and Greco (1961). Since variations in the colour intensities have been suggested by some workers as inherent in the method, the technique was tested and standardized by estimations on collagen and gelatin samples to ensure that the procedures were followed exactly.

Standard curves for the absorption values of the coloured solutions were made for the ranges 10–30 $\mu$g. and 20–50 $\mu$g. hydroxyproline in a final volume of 10 ml. and a standard solution was included in each batch of analyses. The absorptions were measured on a Unicam SP 500 spectrophotometer at 580 m$\mu$.

*Tyrosine.*—The method previously used by Hamer and Marchant (1957) for the tyrosine estimation was adopted, measuring the colour obtained with the 1-nitro-2-naphthol reagent (Udenfriend and Cooper, 1952) at 450 m$\mu$ on hydrolysed samples containing 20–40 $\mu$g. tyrosine in the final 4 ml. aqueous solution from which excess reagent had been extracted with chloroform.

*Cystine.*—For cystine the colorimetric method of Shinohara and Padis (1936) was used employing the phospho-18-tungstic acid reagent of Folin and Marenzi (1929). Solutions containing about 250 $\mu$g. in a final volume of 25 ml. were estimated at 720 m$\mu$.

### RESULTS

In the main series of tests skins from animals with papillomas were not used, neither were any which showed " accidental " damage of any kind. A few tests were carried out on pooled skin from mice with tumours, in some instances multiple, produced by long-term application of the carcinogen.

The values for tyrosine, nitrogen and hydroxyproline given in the following tables, represent the mean values of two analyses on each of two samples. There was no doubt that the fragments of pooled skins had become thoroughly mixed during the extraction procedures. Differences between the results of individual tests were of the same order as found by Hamer and Marchant (1957), namely $\pm 5$ per cent for tyrosine, $\pm 3$ per cent for hydroxyproline and $\pm 3$ per cent for nitrogen. It may be noted at this point that slight differences in the present values from those reported would be expected since the samples Hamer and Marchant analysed contained lipid and fat which were removed by the organic solvents from the specimens used by Fels and Greco (1961) and from those considered in this report. Incidentally it may be remarked that although Fels and Greco's paper is entitled "Changes in epidermal hydroxyproline and tyrosine ", and the legends to their charts read " Changes in epithelial tyrosine etc.", it must be presumed from the methods described that the samples were in fact of whole skin, since there is no suggestion that the epidermis was separated.

### *I. Water and fat content*

The average fat and water content of the skin specimens from 4 groups of untreated male stock white mice, 3 animals in each group, at the commencement of the tests was 80·3 per cent, i.e. 19·7 per cent protein plus substances insoluble in acetone, ether and chloroform, and 82·0 per cent for 2 groups of untreated females, i.e. 18 per cent insoluble material. No significant alterations were found during the treatment with the carcinogens or with the solvents only.

*II. Nitrogen*

The nitrogen contents of the dry skin samples are given in Table II. The average initial value for the albino male groups was 13·8 per cent and for groups of females 13·5 per cent. The values for C3H males from the charts of Fels and Greco (1961) show 15–15·3 mg. per cent N with a slight decrease, in general, as treatment with either benzene or carcinogen solution proceeded. In their carcinogen-treated series there were appreciable falls to approximately 12 mg. per cent at 20 days and at 80 days. The values in all the present series showed less fluctuation, the lowest value being 12·55 mg. per cent after acetone treatment for 70 days.

TABLE II.—*Nitrogen Content of Mouse Skins*
(*Mg. N per* 100 *mg. dry fat-free skin*)

| | Duration of treatment (days) | | | | | | | | | |
|---|---|---|---|---|---|---|---|---|---|---|
| Series | 0 | 10 | 20 | 30 | 40 | 60 | 70 | 80 | 100 | 110 |
| A.I . | — | 13·6 | — | 14·6 | 14·1 | 14·5 | 14·75 | 14·8 | 14·6 | 15·1 |
| A II . | 13·2 | 13·8 | 14·5 | 14·4 | 14·5 | — | 12·55 | 14·0 | 14·65 | 14·7 |
| B.I . | — | 14·0 | 14·55 | 14·6 | 14·7 | 14·3 | 14·7 | 14·9 | 14·7 | 15·0 |
| B.II . | 14·65 | 13·9 | 13·83 | 14·45 | 14·1 | 14·1 | 15·3 | 14·65 | 14·8 | 13·85 |
| C . | 13·6 | 13·7 | 14·3 | 14·3 | 14·9 | 14·9 | 14·4 | 14·35 | 13·05 | 14·85 |
| D.I . | 14·2 | 13·8 | 14·0 | — | 14·1 | 13 9 | 14·2 | 14.0 | — | — |
| D II . | — | 13·6 | 13·8 | — | 13·2 | 13·8 | 14·2 | 13·6 | — | — |
| E . | — | 14·3 | — | 13·9 | 13·6 | 14·2 | 14·9 | 14·5 | 14·0 | 14·1 |
| F . | 13·5 | 13·4 | — | 13·9 | 13·2 | 14·2 | 13·8 | 14·1 | 13·8 | 13·7 |
| G . | — | 14·7 | — | 14·0 | — | 14·25 | — | 14·3 | — | — |

*III. Tyrosine content.*

The values for the three amino acids have been expressed as mg. amino acid nitrogen per 100 mg. of dry, defatted skin (protein) nitrogen.

*Control groups (albino males)*.—The specimens from the groups treated with acetone or benzene showed very little alteration in tyrosine content throughout 16 weeks (Table III). In Fels and Greco's series an increase from 1·0 mg. to as much as 4·4 mg. per 100 mg. protein nitrogen was found. Hamer and Marchant's value of 0·57 mg. is probably somewhat lower than would be given by defatted material. Specimens taken from untreated male animals 8 to 20 weeks old also showed very constant values indicating that little change would be caused by the ageing of the animals during the main experiments.

*Carcinogen treated groups*.—No marked progressive rise in tyrosine was observed during treatment with methylcholanthrene, either 0·3 per cent or 0·6 per cent in benzene or 0·3 per cent in acetone, the values (1·3–1·6 mg. tyrosine N/100 mg. protein N) contrasting with figures of 4 times that amount in Fels and Greco's specimens at 100 days but agreeing with Hamer and Marchant's content of less than 1 mg. for specimens before, or after 12 weeks treatment. The values for

TABLE III.—*Tyrosine Content of Mouse Skin*
*(Mg. Tyrosine/100 mg. dry, fat-free skin Nitrogen)*

| Series | Duration of treatment (days) | | | | | | | | | | |
|---|---|---|---|---|---|---|---|---|---|---|---|
| | 0 | 10 | 20 | 30 | 40 | 60 | 70 | 80 | 90 | 110 | 110 |
| A.I | — | 1·13 | — | 1·06 | 1·2 | 1·3 | 1·3 | 1·7 | — | 1·6 | 1·65 |
| A.II | 1·15 | 0·97 | — | 1·02 | 0·90 | 1·1 | 1·1 | 0·90 | — | 1·20 | 1·39 |
| B.I | — | 1·21 | — | 0·83 | 1·2 | 1·15 | 1·06 | 1·21 | — | 1·28 | 1·33 |
| B.II | 0·84 | 0·79 | 1·0 | 0·82 | 0·69 | 0·88 | 1·0 | 1·12 | — | 1·32 | — |
| C | — | 0·90 | — | 0·84 | 1·0 | 1·2 | 1·05 | 1·13 | — | 1·42 | 1·60 |
| D.I | 1·03 | 1·0 | 1·20 | — | 1·1 | 1·44 | 1·5 | — | — | — | — |
| D.II | 1·02 | 1·0 | 1·3 | — | 1·6 | 1·4 | 1·5 | — | 1·2 | — | — |
| E | — | 0·93 | 1·62 | — | 1·02 | 1·17 | — | 0·89 | 0·93 | — | — |
| F | — | 0·94 | 0·74 | — | 1·18 | 1·1 | — | 1·07 | 1·26 | — | — |
| G | — | 1·33 | — | 0.99 | — | 1·25 | — | 1·20 | — | — | — |

animals treated with the other carcinogens also showed little fluctuation over the whole period.

*IV. Hydroxyproline content*

*Control groups.*—In the stock white mice treated only with the solvents there was very little change in the hydroxyproline values, initially 4·28 and 4·4 mg. per 100 mg. protein nitrogen for males and 3·47 mg. for females (Table IV). One male

TABLE IV.—*Hydroxyproline Content of Mouse Skin*
*(Mg. Hydroxyproline/100 mg. dry, fat-free skin Nitrogen)*

| Series | Duration of treatment (days) | | | | | | | | | | | |
|---|---|---|---|---|---|---|---|---|---|---|---|---|
| | 0 | 10 | 20 | 30 | 40 | 50 | 60 | 70 | 80 | 90 | 100 | 110 |
| A.I | 4·15 | 3·41 | — | 2·5 | 2·75 | 2·5 | 3·6 | 3·3 | — | 2·5 | 2·6 | 2·8 |
| A.II | 4·28 | 4·61 | — | 4·75 | 4·0 | — | 4·1 | 3·2 | 4·0 | — | 4·2 | 4·0 |
| B.I | 4·28 | 3·08 | — | 2·92 | 3.96 | — | 3·5 | 3·2 | 2·7 | — | 3·0 | 2·5 |
| B.II | 4·4 | 4·0 | 4·1 | 4·5 | 4·0 | — | 4·0 | 3·8 | 4·3 | — | 4·1 | — |
| C | 4·28 | 4·06 | — | 3·12 | 3·15 | — | 3·5 | 3·0 | 3·2 | — | 3·8 | 3·4 |
| D.I | — | 4·0 | 2·92 | 3·01 | 2·8 | — | 2·7 | 2·9 | — | — | — | — |
| D.II | 4·96 | 4·5 | 4·6 | — | 3·9 | — | 4·0 | 3·7 | 3·7 | 3·6 | — | — |
| E | — | 3·6 | 3·08 | — | 2·78 | — | 2·8 | — | 2·8 | 3·3 | — | — |
| F | 3·47 | 2·96 | 2·74 | — | 2·51 | — | 2·49 | — | 2·63 | 2·75 | — | — |
| G | — | 3·21 | — | 4·16 | — | — | 3·25 | — | 4·5 | — | — | — |

specimen after acetone treatment for 70 days had 3·2 mg. but values of 4·0–4·3 mg. were otherwise observed.

Also the hydroxyproline content did not alter appreciably in stock white mice between 8 and 20 weeks of age not subjected to any treatment except hair clipping.

*Carcinogen treated groups.*—Values somewhat below those for untreated animals were obtained throughout this series, the lowest being 2·5 mg. per cent or 60 per cent of the value for specimens from untreated animals. This reduction, was however, not so pronounced as in Fels and Greco's experiments, namely a value 45 per cent of the initial 6 mg. per cent content, obtained at 90 days, and the remarkably low figure of 16 per cent of the initial content, obtained at 80 days.

Fels and Greco's value of 6·0 mg. per cent hydroxyproline nitrogen, would seem to be very high compared with Hamer and Marchant's value of 4·2 mg. per cent hydroxyproline nitrogen, and their parallel analyses for collagen extracted from untreated albino skins. However, the value of 4·96 mg. obtained from our untreated C3H specimens was somewhat higher than those for the albino strain.

*V. Cystine content*

The values for cystine (Table V) do not reveal noteworthy changes during any of the treatments. There is a slight trend towards an increase in some of the groups after about 40 days.

TABLE V.—*Cystine Content of Mouse Skin*
(*Mg. Cystine N/100 mg. dry, fat-free skin Nitrogen*)

| | Duration of treatment (days). | | | | | | | | | |
|---|---|---|---|---|---|---|---|---|---|---|
| Series | 0 | 10 | 20 | 30 | 40 | 60 | 70 | 80 | 100 | 110 |
| A.I . | — | 1·16 | 1·20 | 1·10 | 1·15 | 1·74 | 1·81 | 1·46 | 1·45 | 1·35 |
| A.II . | 1·31 | 1·27 | — | 1·33 | — | 1·52 | 1·28 | 1·72 | — | 1·28 |
| B.I . | — | 1·17 | — | 1·34 | 1·32 | — | 1·11 | 1·29 | — | 1·37 |
| B.II . | — | 1·02 | 1·0 | 1·4 | — | 1·70 | 1·6 | — | 1·56 | — |
| C . | 1·15 | 1·00 | 1·16 | 1·07 | — | — | — | 1·22 | 1·68 | 1·83 |
| D.I . | 1·15 | — | — | 1·44 | — | 1·62 | — | 1·57 | — | — |
| D.II . | 1·2 | — | — | 1·33 | — | 1·39 | — | — | 1·37 | — |
| E . | — | 1·03 | — | — | 1·70 | 1·47 | — | — | 1·66 | — |
| F . | 1.20 | — | — | 1.4 | — | 1.45 | — | 1.52 | 1.55 | — |
| G . | — | 1·27 | — | 1·01 | — | 1·32 | — | 1·20 | 1·62 | 1·68 |

*VI. Tumour-bearing skins*

Specimens of pooled albino male mice each bearing a number of small papillomas were found to contain 3·06 mg. hydroxyproline nitrogen and 1·83 mg. tyrosine nitrogen, per 100 mg. protein nitrogen. The former value is close to that found in non-tumour-bearing skin after 80 days carcinogen treatment but the tyrosine content is higher, possibly denoting the increase in keratin to be expected in parts of the surface layer.

*VII. Comparison with C3H strain of mice*

During treatment with benzene there was some fall in the hydroxyproline content of the skins of our C3H strain mice, the values at 70 and 80 days being 77 per cent of the original, but not significantly below those for albinos similarly treated. Methylcholanthrene treatment for this time did not produce values less than those for the albino mice.

### DISCUSSION

The results obtained in this investigation confirm, in general, the previous conclusion of Hamer and Marchant (1957) that during the tumour induction period in male mice there occurs a slight loss of hydroxyproline in the skin protein with little demonstrable change in the tyrosine.

In comparing the results of this and earlier work it should be remembered that the weekly dosage of hydrocarbon delivered to the skin varied. Hamer and Marchant applied 0·9 mg. in one application. Fels and Greco (1961) applied 0·3 mg. in three applications. An intermediate dose of 0·6 mg. per week, given in two applications, was selected here. The difference in results, therefore, would not appear to be due directly to this factor.

However, Fels and Greco stated that in their experiments papillomas developed very early—some time after 2 weeks' applications. Hamer and Marchant found small papillomas at a much later time. Ulceration was also noted by the American workers, which is not usual with the animals of our albino strain during treatment with the carcinogen at the concentration used. Nor did our C3H animals appear to be more sensitive in this respect. While there may be some chemical changes accompanying such rapid proliferation and tumour development, the changes noted by Fels and Greco might be rather a reflection of skin damage and necrosis. In our experience it is unusual to produce papillomas with 0·3 per cent methylcholanthrene earlier than 8 weeks. In the present experiments papillomas were found after 100 days using 0·3 per cent methylcholanthrene in acetone or benzene, while the first papillomas with 0·1 per cent 9,10-dimethyl-1,2-benzanthracene were produced after 60 days. The fact that cystine and tyrosine may be higher in the skin of tumour-bearing mice could be inferred from the well-known hyperkeratosis associated with papillomas and squamous cell carcinomas. This has little relevance to the question of chemical alterations in the preceding period which is of more consequence for understanding carcinogenesis.

### SUMMARY

Solutions of carcinogenic hydrocarbons in acetone or benzene were applied twice weekly to the skins of albino and C3H mice. Samples of the treated skins, free of macroscopic tumours were taken at frequent intervals, up to 16 weeks. The total nitrogen, hydroxyproline, tyrosine and cystine contents of hydrolysates of the dry fat-free specimens were determined.

The values for hydroxyproline nitrogen in terms of total skin nitrogen showed a slight decrease during the treatment period. The content of tyrosine and cystine did not alter appreciably.

This work was carried out with the financial support of the Birmingham Branch of the British Empire Cancer Campaign.

### REFERENCES

Fels, I. G. and Greco, J.—(1961) *Cancer Res.*, **21**, 40.
*Idem* and Veach, R.—(1959) *Analyt. Chem.*, **31**, 451.
Folin, O. and Marenzi, A. D.—(1929) *J. biol. Chem.*, **83**, 109.
Hamer, D. and Marchant, J.—(1957) *Brit. J. Cancer*, **11**, 445.
Neuman, R. E. and Logan, M. A.—(1950) *J. biol. Chem.*, **184**, 299.
Orr, J. W.—(1938) *J. Path. Bact.*, **46**, 495.
Shinohara, K. and Padis, K. E.—(1936) *J. biol. Chem.*, **112**, 714.
Udenfriend, S. and Cooper, J. R.—(1952) *Ibid.*, **196**, 227.
Vernoni, G.—(1951) *Sci. Med. Ital.*, **2**, 369.